# On *p*-radial Blaschke and harmonic Blaschke additions

**DOI:** 10.1186/s13660-017-1581-y

**Published:** 2017-12-16

**Authors:** Chang-Jian Zhao

**Affiliations:** Department of Mathematics, China Jiliang University, Hangzhou, 310018 P.R. China

**Keywords:** 46E27, 52A20, radial Blaschke addition, harmonic Blaschke addition, *p*-radial Blaschke addition, *p*-harmonic Blaschke addition, radial Blaschke-Minkowski homomorphisms, Brunn-Minkowski inequality

## Abstract

In the paper, we first improve the radial Blaschke and harmonic Blaschke additions and introduce the *p*-radial Blaschke and *p*-harmonic Blaschke additions. Following this, Dresher type inequalities for the radial Blaschke-Minkowski homomorphisms with respect to *p*-radial Blaschke and *p*-harmonic Blaschke additions are established.

## Notation and preliminaries

The setting for this paper is an *n*-dimensional Euclidean space ${\Bbb {R}}^{n}$. We reserve the letter *u* for unit vectors, and the letter *B* is reserved for the unit ball centered at the origin. The surface of *B* is $S^{n-1}$. The volume of the unit *n*-ball is denoted by $\omega_{n}$. We use $V(K)$ for the *n*-dimensional volume of a body *K*. Associated with a compact subset *K* of ${\Bbb {R}}^{n}$, which is star-shaped with respect to the origin, is its radial function $\rho(K,\cdot): S^{n-1}\rightarrow{\Bbb {R}}$ defined for $u\in S^{n-1}$ by

$$\rho(K,u)=\max\{\lambda\geq0: \lambda u\in K\}. $$

If $\rho(K,\cdot)$ is positive and continuous, *K* will be called a star body. Let ${\mathcal {S}}^{n}$ denote the set of star bodies in ${\Bbb {R}}^{n}$. Let *δ̃* denote the radial Hausdorff metric, i.e., if $K, L\in{\mathcal {S}}^{n}$, then $\tilde{\delta}(K,L)=|\rho(K,u)-\rho(L,u)|_{\infty}$, where $|\cdot|_{\infty}$ denotes the sup-norm on the space of continuous functions $C(S^{n-1})$.

### Dual mixed volumes

The radial Minkowski linear combination, $\lambda_{1}K_{1}\widetilde {+}\cdots\widetilde{+}\lambda_{r} K_{r}$ is defined by

1.1 $$\begin{aligned} \lambda_{1}K_{1}\widetilde{+}\cdots\widetilde{+} \lambda_{r} K_{r}=\{\lambda_{1}x_{1} \widetilde{+}\cdots\widetilde{+}\lambda_{r} x_{r}: x_{i}\in K_{i}, i=1,\ldots,r\} \end{aligned}$$

for $K_{1},\ldots,K_{r}\in{\mathcal {S}}^{n}$ and $\lambda_{1},\ldots,\lambda_{r}\in{\Bbb {R}}$. It has the following important property (see [1]):

1.2 $$\begin{aligned} \rho(\lambda K\widetilde{+}\mu L,\cdot)=\lambda\rho(K,\cdot)+\mu\rho(L, \cdot) \end{aligned}$$

for $K, L\in{\mathcal {S}}^{n}$ and $\lambda, \mu\geq0$. For $K_{1},\ldots,K_{r}\in{\mathcal {S}}^{n}$ and $\lambda_{1},\ldots,\lambda_{r}\geq0$, the volume of the radial Minkowski linear combination $\lambda_{1}K_{1}\widetilde{+}\cdots\widetilde{+}\lambda_{r}K_{r}$ is a homogeneous polynomial of degree *n* in the $\lambda_{i}$,

1.3 $$\begin{aligned} V(\lambda_{1}K_{1}\widetilde{+}\cdots\widetilde{+}\lambda _{r}K_{r})=\sum_{i_{1},\ldots,i_{n}=1}^{r} \widetilde{V}(K_{i_{1}},\ldots,K_{i_{n}})\lambda_{i_{1}} \cdots \lambda_{i_{n}}. \end{aligned}$$

If we require the coefficients of the polynomial in (1.3) to be symmetric in their arguments, then they are uniquely determined. The coefficient $\widetilde{V}(K_{i_{1}},\ldots,K_{i_{n}})$ is nonnegative and depends only on the bodies $K_{i_{1}},\ldots,K_{i_{n}}$. It is called the dual mixed volume of $K_{i_{1}},\ldots,K_{i_{n}}$.

If $K_{1},\ldots,K_{n}\in{\mathcal {S}}^{n}$, then the dual mixed volume $\widetilde{V}(K_{1},\ldots,K_{n})$ can be represented in the form (see [2])

1.4 $$\begin{aligned} \widetilde{V}(K_{1},\ldots,K_{n})=\frac{1}{n} \int_{S^{n-1}}\rho (K_{1},u)\cdots\rho(K_{n},u)\,dS(u). \end{aligned}$$

If $K_{1}=\cdots=K_{n-i}=K$, $K_{n-i+1}=\cdots=K_{n}=L$, then the dual mixed volume is written as $\widetilde{V}_{i}(K,L)$. If $L=B$, then the dual mixed volume $\widetilde{V}_{i}(K,L)=\widetilde{V}_{i}(K,B)$ is written as $\widetilde{W}_{i}(K)$. For $K,L\in{\mathcal {S}}^{n}$, the *i*th dual mixed volume of *K* and *L*, $\widetilde{V}_{i}(K,L)$ can be extended to all $i\in{\Bbb {R}}$ by

1.5 $$\begin{aligned} \widetilde{V}_{i}(K,L)=\frac{1}{n} \int_{S^{n-1}}\rho(K,u)^{n-i}\rho (L,u)^{i}\,dS(u), \end{aligned}$$

where $i\in{\Bbb {R}}$. Thus, if $K\in{\mathcal {S}}^{n}$, then

1.6 $$\begin{aligned} \widetilde{W}_{i}(K)=\frac{1}{n} \int_{S^{n-1}}\rho (K,u)^{n-i}\,dS(u). \end{aligned}$$

### Mixed intersection bodies

For $K\in{\mathcal {S}}^{n}$, there is a unique star body **I** *K* whose radial function satisfies, for $u\in S^{n-1}$,

$$\rho(\mathbf{I}K,u)=v(K\cap E_{u}), $$

where *v* is $(n-1)$-dimensional dual volume. It is called the intersection body of *K*. The volume of the intersection body of *K* is given by (see [1])

$$V(\mathbf{I}K)=\frac{1}{n} \int_{S^{n-1}}v(K\cap E_{u})^{n}\,dS(u). $$

The mixed intersection body of $K_{1},\ldots,K_{n-1}\in{\mathcal {S} }^{n}$, denoted by $\mathbf{I}(K_{1},\ldots,K_{n-1})$, is defined by

$$\rho\bigl(\mathbf{I}(K_{1},\ldots,K_{n-1}),u\bigr)= \tilde{v}(K_{1}\cap E_{u},\ldots,K_{n-1}\cap E_{u}), $$

where *ṽ* is the $(n-1)$-dimensional dual mixed volume. If $K_{1}=\cdots=K_{n-i-1}=K, K_{n-i}=\cdots=K_{n-1}=L$, then ${\bf I}(K_{1},\ldots,K_{n-1})$ is written as $\mathbf{I}_{i}(K,L)$. If $L=B$, then $\mathbf{I}_{i}(K,L)$ is written as $\mathbf{I}_{i}K$ and called the *i*th intersection body of *K*. For $\mathbf{I}_{0}K$, we simply write **I** *K*.

## Improvement of the radial Blaschke addition

Let us recall the concept of radial Blaschke addition defined by Lutwak [1]. Suppose that *K* and *L* are star bodies in ${\Bbb {R}}^{n}$, the radial Blaschke addition denoted by $K\widehat{+}L$ is a star body whose radial function is

2.1 $$\begin{aligned} \rho(K\widehat{+}L, \cdot)^{n-1}=\rho(K,\cdot)^{n-1}+\rho(L, \cdot)^{n-1}. \end{aligned}$$

The dual Knesser-Süss inequality for the radial Blaschke addition was established by Lutwak [1]. If $K,L\in{\mathcal {S}}^{n}$, then

2.2 $$\begin{aligned} V(K\widehat{+}L)^{(n-1)/n}\leq V(K)^{(n-1)/n}+V(L)^{(n-1)/n}, \end{aligned}$$

with equality if and only if *K* and *L* are dilates.

In the section, we give a generalized concept of the radial Blaschke addition.

### Definition 2.1

If $K,L\in{\mathcal {S}}^{n}$, $0\leq p< n-1$ and $\lambda, \mu>0$ (not both zero), the *p*-radial Blaschke linear combination of *K* and *L* denoted by $\lambda\diamond K\widehat{+}_{p}\mu\diamond L$ is a star body whose radial function is defined by

2.3 $$\begin{aligned} \rho(\lambda\diamond K\widehat{+}_{p}\mu\diamond L,\cdot )^{n-p-1}=\lambda\rho(K,\cdot)^{n-p-1}+\mu\rho(L,\cdot )^{n-p-1}. \end{aligned}$$

From (2.3), it is easy to see that

$$\lambda\diamond K=\lambda^{1/(n-p-1)} K. $$

When $\lambda=\mu=1$, the *p*-radial Blaschke combination becomes the *p*-radial Blaschke addition $K\widehat{+}_{p}L$ and

2.4 $$\begin{aligned} \rho(K\widehat{+}_{p}L,\cdot)^{n-p-1}=\rho(K, \cdot)^{n-p-1}+\rho (L,\cdot)^{n-p-1}. \end{aligned}$$

Obviously, when $p=0$, (2.4) becomes (2.1).

In the following, we define the dual mixed quermassintegral with respect to the *p*-radial Blaschke addition. First, we show two propositions. The following proposition follows immediately from (2.3) with L’Hôpital’s rule.

### Proposition 2.2

*Let* $0\leq p< n-1$, $0\leq i< n$ *and* $\varepsilon>0$. *If* $K,L\in{\mathcal {S}}^{n}$, *then*

2.5 $$\begin{aligned} \lim_{\varepsilon\rightarrow0^{+}}\frac{\rho(K\widehat {+}_{p}\varepsilon\diamond L,u)^{n-i}-\rho(K,u)^{n-i}}{\varepsilon}=\frac{n-i}{n-p-1}\rho (K,u)^{p-i+1}\rho(L,u)^{n-p-1}. \end{aligned}$$

The following proposition follows immediately from Proposition 2.2 and (1.6).

### Proposition 2.3

*Let* $0\leq p< n-1$, $0\leq i< n$ *and* $\varepsilon>0$. *If* $K,L\in{\mathcal {S}}^{n}$, *then*

2.6 $$\begin{aligned} &\frac{n-p-1}{n-i}\lim_{\varepsilon\rightarrow0^{+}}\frac {\widetilde{W}_{i}(K\widehat{+}_{p}\varepsilon\diamond L,u)-\widetilde{W}_{i}(K)}{\varepsilon} \\ &\quad=\frac{1}{n} \int _{S^{n-1}}\rho(K,u)^{p-i+1}\rho(L,u)^{n-p-1}\,dS(u). \end{aligned}$$

### Definition 2.4

For $0\leq p< n-1$, $0\leq i< n$ and $K,L\in {\mathcal {S}}^{n}$, the *p*-dual mixed quermassintegral of star bodies *K* and *L*, denoted by $\widetilde{W}_{p,i}(K,L)$, is defined by

2.7 $$\begin{aligned} \widetilde{W}_{p,i}(K,L)=\frac{1}{n} \int_{S^{n-1}}\rho (K,u)^{p-i+1}\rho(L,u)^{n-p-1}\,dS(u). \end{aligned}$$

Obviously, when $K=L$, $\widetilde{W}_{p,i}(K,L)$ becomes the dual quermassintegral of star body *K*, i.e., $\widetilde{W}_{p,i}(K,K)=\widetilde{W}_{i}(K)$. Taking $i=0$ in (2.7), $\widetilde{W}_{p,i}(K,L)$ becomes the *p*-dual mixed volume $\widetilde{V}_{p}(K,L)$ and

2.8 $$\begin{aligned} \widetilde{V}_{p}(K,L)=\frac{1}{n} \int_{S^{n-1}}\rho(K,u)^{p+1}\rho (L,u)^{n-p-1}\,dS(u). \end{aligned}$$

From (2.7), combining Hölder’s integral inequality (see [3]) gives the following.

### Proposition 2.5

(Minkowski type inequality)

*If* $K,L\in{\mathcal {S}}^{n}$, $0\leq i< n$ *and* $0\leq p< n-1$, *then*

2.9 $$\begin{aligned} \widetilde{W}_{p,i}(K,L)^{n-i}\leq\widetilde {W}_{i}(K)^{p-i+1}\widetilde{W}_{i}(L)^{n-p-1}, \end{aligned}$$

*with equality if and only if* *K* *and* *L* *are dilates*.

Taking $i=0$ in (2.9), we have: If $K,L\in{\mathcal {S}}^{n}$ and $0\leq p< n-1$, then

2.10 $$\begin{aligned} \widetilde{V}_{p}(K,L)^{n}\leq V(K)^{p+1}V(L)^{n-p-1}, \end{aligned}$$

with equality if and only if *K* and *L* are dilates. In the following, we establish the Brunn-Minkowski inequality for the *p*-radial Blaschke addition.

### Proposition 2.6

*If* $K,L\in{\mathcal {S}}^{n}$, $0\leq i< n$ *and* $0\leq p< n-1$, *then*

2.11 $$\begin{aligned} \widetilde{W}_{i}(K\widehat{+}_{p}L)^{(n-p-1)/(n-i)}\leq \widetilde {W}_{i}(K)^{(n-p-1)/(n-i)}+\widetilde{W}_{i}(L)^{(n-p-1)/(n-i)}, \end{aligned}$$

*with equality if and only if* *K* *and* *L* *are dilates*.

### Proof

From (2.3) and (2.7), it is easily seen that the *p*-dual mixed quermassintegral $\widehat{W}_{p,i}(K,L)$ is linear with respect to the *p*-radial Blaschke addition and together with inequality (2.9) shows that

2.12 $$\begin{aligned} \widetilde{W}_{p,i}(Q, K\widehat{+}_{p}L)&=\widetilde {W}_{p,i}(Q,K)+\widetilde {W}_{p,i}(Q,L) \\ &\leq\widetilde{W}_{i}(Q)^{(p-i+1)/(n-i)}\bigl(\widetilde {W}_{i}(K)^{(n-p-1)/(n-i)}+\widetilde {W}_{i}(L)^{(n-p-1)/(n-i)} \bigr), \end{aligned}$$

with equality if and only if *K* and *L* are dilates of *Q*. Take $K\widehat{+}_{p}L$ for *Q* in (2.12), recall that $\widetilde{W}_{p,i}(Q,Q)=\widetilde{W}_{i}(Q)$, inequality (2.11) follows easy.

Taking $i=0$ in (2.11), we obtain that if $K,L\in{\mathcal {S}}^{n}$ and $0\leq p< n-1$, then

$$V(K\widehat{+}_{p} L)^{(n-p-1)/n}\leq V(K)^{(n-p-1)/n}+V(L)^{(n-p-1)/n}, $$

with equality if and only if *K* and *L* are dilates. Taking $p=0$ and $i=0$ in (2.11), (2.11) becomes the well-known dual Knesser-Süss inequality (2.2). □

## Improvement of the harmonic Blaschke addition

Let us recall the concept of harmonic Blaschke addition defined by Lutwak [4]. Suppose that *K* and *L* are star bodies in ${\Bbb {R}}^{n}$, the harmonic Blaschke addition denoted by $K\breve{+}L$ is defined by

3.1 $$\begin{aligned} \frac{\rho(K\breve{+}L, \cdot)^{n+1}}{V(K\breve{+}L)}=\frac{\rho(K,\cdot )^{n+1}}{V(K)}+\frac{\rho(L,\cdot)^{n+1}}{V(L)}. \end{aligned}$$

Lutwak’s Brunn-Minkowski inequality for the harmonic Blaschke addition was established (see [4]). If $K,L\in{\mathcal {S}}^{n}$, then

3.2 $$\begin{aligned} V(K\breve{+}L)^{1/n}\geq V(K)^{1/n}+V(L)^{1/n}, \end{aligned}$$

with equality if and only if *K* and *L* are dilates.

In the section, we give an improved concept of the harmonic Blaschke addition.

### Definition 3.1

For $0\leq i< n$, $p< i-1$ and $K,L\in{\mathcal {S}}^{n}$, we define the *p*-harmonic Blaschke addition of star bodies *K* and *L* denoted by $K\breve{+}_{p}L$ and defined by

3.3 $$\begin{aligned} \frac{\rho(K\breve{+}_{p}L,\cdot)^{n-p-1}}{\tilde{W}_{i}(K\breve {+}_{p}L)}=\frac{\rho(K,\cdot)^{n-p-1}}{ \widetilde{W}_{i}(K)}+\frac{\rho(L,\cdot)^{n-p-1}}{\tilde {W}_{i}(L)}. \end{aligned}$$

The Brunn-Minkowski inequality for the *p*-harmonic Blaschke addition follows immediately from (1.6), (3.3) and Minkowski’s integral inequality (see [3]).

### Proposition 3.2

*If* $K,L\in{\mathcal {S}}^{n}$, $0\leq i< n$ *and* $p< i-1$, *then*

3.4 $$\begin{aligned} \widetilde{W}_{i}(K\breve{+}_{p}L)^{-(p+1-i)/(n-i)}\leq \widetilde {W}_{i}(K)^{-(p+1-i)/(n-i)} +\widetilde{W}_{i}(L)^{-(p+1-i)/(n-i)}, \end{aligned}$$

*with equality if and only if* *K* *and* *L* *are dilates*.

## Radial Blaschke-Minkowski homomorphisms

### Definition 4.1

([5])

A map $\Psi: {\mathcal {S}}^{n}\rightarrow {\mathcal {S}}^{n}$ is called a radial Blaschke-Minkowski homomorphism if it satisfies the following conditions:

- Ψ is continuous.
- For all $K,L\in{\mathcal {S}}^{n}$,
  $$\Psi(K\ddot{+}L)=\Psi(K)\widetilde{+}\Psi(L). $$
- For all $K,L\in{\mathcal {S}}^{n}$ and every $\vartheta\in SO(n)$,
  $$\Psi(\vartheta K)=\vartheta\Psi(K), $$
  where $SO(n)$ is the group of rotations in *n* dimensions.

Radial Blaschke-Minkowski homomorphisms are important examples of star body valued valuations. Their natural duals, Blaschke-Minkowski homomorphisms, are an important notion in the theory of convex body valued valuations (see, e.g., [6–12] and [13–20]). In 2006, Schuster [5] established the following Brunn-Minkowski inequality for radial Blaschke-Minkowski homomorphisms of star bodies. If *K* and *L* are star bodies in ${\Bbb {R}}^{n}$, then

4.1 $$\begin{aligned} V\bigl(\Psi(K\widetilde{+}L)\bigr)^{1/n(n-1)}\leq V(\Psi K)^{1/n(n-1)}+V(\Psi L)^{1/n(n-1)}, \end{aligned}$$

with equality if and only if *K* and *L* are dilates.

If *K* and *L* are star bodies in ${\Bbb {R}}^{n}$, $p\neq0$ and $\lambda, \mu\geq0$, then $\lambda\cdot K\widetilde{+}_{p}\mu\cdot L$ is the star body whose radial function is given by (see, e.g., [21])

4.2 $$\begin{aligned} \rho(\lambda\cdot K\widetilde{+}_{p}\mu\cdot L,\cdot)^{p}= \lambda\rho(K,\cdot)^{p}+\mu\rho(L,\cdot )^{p}. \end{aligned}$$

The addition $\widetilde{+}_{p}$ is called $L_{p}$-radial addition. The $L_{p}$ dual Brunn-Minkowski inequality states: If $K,L\in{\mathcal {S}}^{n}$ and $0< p\leq n$, then

$$V(K\widetilde{+}_{p}L)^{p/n}\leq V(K)^{p/n}+V(L)^{p/n}, $$

with equality when $p\neq n$ if and only if *K* and *L* are dilates. The inequality is reversed when $p>n$ or $p<0$ (see [21]).

In 2013, an $L_{p}$ Brunn-Minkowski inequality for radial Blaschke-Minkowski homomorphisms was established in [22]: If *K* and *L* are star bodies in ${\Bbb {R}}^{n}$ and $0< p< n-1$, then

4.3 $$\begin{aligned} V\bigl(\Psi(K\widetilde{+}_{p}L)\bigr)^{p/n(n-1)}\leq V(\Psi K)^{p/n(n-1)}+V(\Psi L)^{p/n(n-1)}, \end{aligned}$$

with equality if and only if *K* and *L* are dilates. Taking $p=1$, (4.3) reduces to (4.1).

### Theorem 4.2

(see [5])

*Let* $\Psi: {\mathcal {S}}^{n}\rightarrow{\mathcal {S}}^{n}$ *be a radial Blaschke*-*Minkowski homomorphism*. *There is a continuous operator* $\Psi: \underbrace{{{\mathcal {S}}^{n}\times\cdots\times\mathcal {S}}^{n}}_{n-1}\rightarrow{\mathcal {S}}^{n}$ *symmetric in its arguments such that*, *for* $K_{1},\ldots,K_{m}\in{\mathcal {S}}^{n}$ *and* $\lambda_{1},\ldots,\lambda_{m}\geq0$,

4.4 $$\begin{aligned} \Psi(\lambda_{1}K_{1}\widetilde{+}\cdots\widetilde{+} \lambda _{m}K_{m})=\sum_{i_{1},\ldots,i_{n-1}} \lambda_{i_{1}}\cdots\lambda _{i_{n-1}} \Psi(K_{i_{1}}, \ldots,K_{i_{n-1}}). \end{aligned}$$

Clearly, Theorem 4.2 generalizes the notion of radial Blaschke-Minkowski homomorphisms. We call $\Psi: {\mathcal {S}}^{n}\times\cdots\times{\mathcal {S}}^{n}\rightarrow{\mathcal {S}}^{n}$ a mixed radial Blaschke-Minkowski homomorphism induced by Ψ. Mixed radial Blaschke-Minkowski homomorphisms were first studied in more detail in [23, 24]. If $K_{1}=\cdots=K_{n-i-1}=K, K_{n-i}=\cdots=K_{n-1}=L$, we write $\Psi_{i}(K,L)$ for $\Psi(\underbrace{K,\ldots,K}_{n-i-1},\underbrace{L,\ldots,L}_{i})$. If $K_{1}=\cdots=K_{n-i-1}=K, K_{n-i}=\cdots=K_{n-1}=B$, we write $\Psi_{i} K$ for $\Psi(\underbrace{K,\ldots,K}_{n-i-1},\underbrace{B,\ldots,B}_{i})$ and call $\Psi_{i} K$ the mixed Blaschke-Minkowski homomorphism of order *i* of *K*. $\Psi_{0} K$ is written simply as Ψ*K*.

### Lemma 4.3

(see [5])

*A map* $\Psi: {\mathcal {S}}^{n}\rightarrow{\mathcal {S}}^{n}$ *is a radial Blaschke*-*Minkowski homomorphism if and only if there is a measure* $\mu\in{\mathcal {M}}_{+}(S^{n-1},\hat{e})$ *such that*

4.5 $$\begin{aligned} \rho(\Psi K,\cdot)=\rho(K,\cdot)^{n-1}\ast\mu, \end{aligned}$$

*where* ${\mathcal {M}}_{+}(S^{n-1},\hat{e})$ *denotes the set of nonnegative zonal measures on* $S^{n-1}$.

For the mixed radial Blaschke-Minkowski homomorphism induced by Ψ, Schuster [5] proved that

$$\rho\bigl(\Psi(K_{1},\ldots,K_{n-1}),\cdot\bigr)= \rho(K_{1},\cdot)\cdots \rho(K_{n-1},\cdot)\ast\mu. $$

Obviously, a special case is the following:

$$\rho(\Psi_{i}K,\cdot)=\rho(K,\cdot)^{n-1-i}\ast\mu, $$

where *i* are integers. We now extend the integers *i* to real numbers, define the Blaschke-Minkowski homomorphism of order *p* of *K*.

### Definition 4.4

Let $K\in{\mathcal {S}}^{n}$, the Blaschke-Minkowski homomorphism of order *p* of *K*, denoted by $\Psi_{p}K$, is defined for all $p\in{\Bbb {R}}$ by

4.6 $$\begin{aligned} \rho(\Psi_{p}K,\cdot)=\rho(K,\cdot)^{n-1-p}\ast \mu. \end{aligned}$$

This extended definition will be required to prove our main results.

## Inequalities for the radial Blaschke-Minkowski homomorphism

### Theorem 5.1

*Let* $K,L\in{\mathcal {S}}^{n}$. *If* $0\leq p< n-1$ *and* $i\leq n-1\leq j\leq n$, *then*

5.1 $$\begin{aligned} \biggl(\frac{\widetilde{W}_{i}(\Psi_{p}(K\widehat {+}_{p}L))}{\widetilde{W}_{j}(\Psi_{p}(K\widehat{+}_{p}L))} \biggr)^{1/(j-i)} \leq \biggl(\frac{\widetilde{W}_{i}(\Psi_{p}K)}{\widetilde {W}_{j}(\Psi_{p}K)} \biggr) ^{1/(j-i)}+ \biggl(\frac{\widetilde{W}_{i}(\Psi_{p} L)}{\widetilde{W}_{j}(\Psi_{p}L)} \biggr)^{1/(j-i)}, \end{aligned}$$

*with equality if and only if* $\Psi_{p} K$ *and* $\Psi_{p} L$ *are dilates*.

### Remark 5.2

Taking $j=n$ in (5.1) and noting that $\widetilde{W}_{n}(K)=\int_{S^{n-1}}\,dS(u)=n\omega_{n}$, (5.1) becomes the following inequality: If $K,L\in{\mathcal {S}}^{n}$, $0\leq p< n-1$ and $i\leq n-1$, then

5.2 $$\begin{aligned} \widetilde{W}_{i}\bigl(\Psi_{p}(K\widehat{+}_{p}L) \bigr)^{1/(n-i)} \leq\widetilde{W}_{i}(\Psi_{p} K)^{1/(n-i)}+\widetilde{W}_{i}(\Psi_{p}L)^{1/(n-i)}, \end{aligned}$$

with equality if and only if $\Psi_{p} K$ and $\Psi_{p} L$ are dilates. Taking $p=0$ in (5.1), (5.1) becomes the following inequality: If $K,L\in{\mathcal {S}}^{n}$ and $i\leq n-1\leq j\leq n$, then

5.3 $$\begin{aligned} \biggl(\frac{\widetilde{W}_{i}(\Psi(K\widehat{+}L))}{\widetilde {W}_{j}(\Psi(K\widehat{+}L))} \biggr)^{1/(j-i)} \leq \biggl(\frac{\widetilde{W}_{i}(\Psi K)}{\widetilde{W}_{j}(\Psi K)} \biggr)^{1/(j-i)}+ \biggl(\frac{\widetilde{W}_{i}(\Psi L)}{\widetilde{W}_{j}(\Psi L)} \biggr)^{1/(j-i)}, \end{aligned}$$

with equality if and only if Ψ*K* and Ψ*L* are dilates.

### Theorem 5.3

*Let* $K,L\in{\mathcal {S}}^{n}$. *If* $0\leq i< n $, $p< i-1$ *and* $k,j\in{\Bbb {R}}$ *satisfy* $j\leq n-1\leq k\leq n$, *then*

5.4 $$\begin{aligned} &\frac{1}{\widetilde{W}_{i}(K\breve{+}_{p} L)} \biggl(\frac{\widetilde{W}_{j}(\Psi_{p}(K\breve {+}_{p}L))}{\widetilde{W}_{k}(\Psi_{p}(K\breve{+}_{p}L))} \biggr)^{1/(k-j)} \\ &\quad\leq\frac{1}{\widetilde{W}_{i}(K)} \biggl(\frac {\widetilde{W}_{j}(\Psi_{p}K)}{\widetilde{W}_{k}(\Psi_{p}K)} \biggr) ^{1/(k-j)}+ \frac{1}{\widetilde{W}_{i}(L)} \biggl(\frac{\widetilde {W}_{j}(\Psi_{p}L)}{\widetilde{W}_{k}(\Psi_{p}L)} \biggr)^{1/(k-j)}, \end{aligned}$$

*with equality if and only if* $\Psi_{p}K$ *and* $\Psi_{p}L$ *are dilates*.

### Remark 5.4

Taking $k=n$ in (5.4) and noting that $\widetilde{W}_{n}(K)=\int_{S^{n-1}}\,dS(u)=n\omega_{n}$, (5.4) becomes the following inequality: If $K,L\in{\mathcal {S}}^{n}$, $0\leq i< n$, $p< i-1$ and $j\leq n-1$, then

5.5 $$\begin{aligned} \frac{\widetilde{W}_{j}(\Psi_{p}(K\breve {+}_{p}L))^{1/(n-j)}}{\widetilde{W}_{i}(K\breve{+}_{p} L)}\leq\frac{\widetilde{W}_{j}(\Psi_{p}K)^{1/(n-j)}}{\widetilde {W}_{i}(K)}+\frac{\widetilde{W}_{j}(\Psi_{p}L)^{1/(n-j)}}{\widetilde {W}_{i}(L)}, \end{aligned}$$

with equality if and only if $\Psi_{p}K$ and $\Psi_{p}L$ are dilates. Taking $i=0$, $j=0$ and $k=n$ in (5.4), we have: If $K,L\in{\mathcal {S}}^{n}$ and $p<-1$, then

5.6 $$\begin{aligned} \frac{V(\Psi_{p}(K\breve{+}_{p}L))^{1/n}}{V(K\breve{+}_{p} L)}\leq\frac{V(\Psi_{p}K)^{1/n}}{V(K)}+\frac{V(\Psi _{p}L)^{1/n}}{V(L)}, \end{aligned}$$

with equality if and only if $\Psi_{p}K$ and $\Psi_{p}L$ are dilates.

## Dresher’s inequalities for *p*-radial Blaschke and harmonic Blaschke additions

An extension of Beckenbach’s inequality (see [3], p. 27) was obtained by Dresher [25] by means of moment-space techniques.

### Lemma 6.1

(Dresher’s inequality)

*If* $p\geq1\geq r\geq0$, $f,g\geq0$ *and* *ϕ* *is a distribution function*, *then*

6.1 $$\begin{aligned} \biggl(\frac{\int(f+g)^{p}\,d\phi}{\int(f+g)^{r}\,d\phi} \biggr)^{1/(p-r)}\leq \biggl(\frac{\int f^{p}\,d\phi}{\int f^{r}\,d\phi} \biggr) ^{1/(p-r)}+ \biggl(\frac{\int g^{p}\,d\phi}{\int g^{r}\,d\phi} \biggr)^{1/(p-r)}, \end{aligned}$$

*with equality if and only if the functions* *f* *and* *g* *are proportional*.

We are now in a position to prove Theorem 5.1. The following statement is just a slight reformulation of it.

### Theorem 6.2

*Let* $K,L\in{\mathcal {S}}^{n}$. *If* $0\leq p< n-1$ *and* $s,t\in{\Bbb {R}}$ *satisfy* $s\geq1\geq t\geq0$, *then*

6.2 $$\begin{aligned} \biggl(\frac{\widetilde{W}_{n-s}(\Psi_{p}(K\widehat {+}_{p}L))}{\widetilde{W}_{n-t}(\Psi_{p}(K\widehat{+}_{p}L))} \biggr)^{1/(s-t)} \leq \biggl(\frac{\widetilde{W}_{n-s}(\Psi_{p}K)}{\widetilde {W}_{n-t}(\Psi_{p}K)} \biggr) ^{1/(s-t)}+ \biggl(\frac{\widetilde{W}_{n-s}(\Psi_{p}L)}{\widetilde {W}_{n-t}(\Psi_{p}L)} \biggr)^{1/(s-t)}, \end{aligned}$$

*with equality if and only if* $\Psi_{p} K$ *and* $\Psi_{p} L$ *are dilates*.

### Proof

From (2.4), we obtain

$$\rho(K\widehat{+}_{p} L,\cdot)^{n-p-1}\ast\mu=\rho(K, \cdot)^{n-p-1}\ast\mu+\rho (L,\cdot)^{n-p-1}\ast\mu, $$

where *μ* is the generating measure of Ψ from Lemma 4.3. Hence, from (4.6), we obtain

$$\rho\bigl(\Psi_{p}(K\widehat{+}_{p}L),\cdot\bigr)=\rho( \Psi_{p}K,\cdot )+\rho(\Psi_{p}L,\cdot). $$

Therefore, by (1.6), we have

6.3 $$\begin{aligned} \widetilde{W}_{n-s}\bigl(\Psi_{p}(K\widehat{+}_{p}L) \bigr)=\frac{1}{n} \int _{S^{n-1}} \bigl(\rho(\Psi_{p}K,u)+\rho( \Psi_{p}L,u) \bigr)^{s}\,dS(u) \end{aligned}$$

and

6.4 $$\begin{aligned} \widetilde{W}_{n-t}\bigl(\Psi_{p}(K\widehat{+}_{p}L) \bigr)=\frac{1}{n} \int _{S^{n-1}} \bigl(\rho(\Psi_{p}K,u)+\rho( \Psi_{p}L,u) \bigr)^{t}\,dS(u). \end{aligned}$$

From (6.3), (6.4) and Lemma 6.1, we obtain

$$\begin{aligned} &\biggl(\frac{\widetilde{W}_{n-s}(\Psi_{p}(K\widehat {+}_{p}L))}{\widetilde{W}_{n-t}(\Psi_{p}(K\widehat{+}_{p}L))} \biggr)^{1/(s-t)} \\ &\quad= \biggl(\frac{\int_{S^{n-1}} (\rho(\Psi_{p}K,u)+\rho(\Psi _{p}L,u) )^{s}\,dS(u)}{ \int_{S^{n-1}} (\rho(\Psi_{p}K,u)+\rho(\Psi_{p}L,u) )^{t}\,dS(u)} \biggr)^{1/(s-t)} \\ &\quad\leq \biggl(\frac{\int_{S^{n-1}}\rho (\Psi_{p}K,u)^{s}\,dS(u)}{ \int_{S^{n-1}}\rho(\Psi_{p}K,u)^{t}\,dS(u)} \biggr)^{1/(s-t)}+ \biggl(\frac {\int_{S^{n-1}}\rho(\Psi_{p}L,u)^{s}\,dS(u)}{\int_{S^{n-1}}\rho(\Psi _{p}L,u)^{t}\,dS(u)} \biggr)^{1/(s-t)} \\ &\quad= \biggl(\frac{\widetilde{W}_{n-s}(\Psi_{p}K)}{\widetilde {W}_{n-t}(\Psi_{p}K)} \biggr) ^{1/(s-t)}+ \biggl(\frac{\widetilde{W}_{n-s}(\Psi_{p}L)}{\widetilde {W}_{n-t}(\Psi_{p}L)} \biggr)^{1/(s-t)}. \end{aligned}$$

Equality holds if and only if the functions $\rho(\Psi_{p}K,u)$ and $\rho(\Psi_{p}L,u)$ are proportional.

Taking $s=n-i$ and $t=n-j$ in Theorem 6.2, Theorem 6.2 becomes Theorem 5.1 stated in Section 5. If $\Psi:\underbrace{{\mathcal {S}}^{n}\times\cdots\times{\mathcal {S}}^{n}}_{n-1}\rightarrow {\mathcal {S}}^{n}$ is the mixed intersection operator ${\bf I}:\underbrace{{\mathcal {S}}^{n}\times\cdots\times{\mathcal {S}}^{n}}_{n-1}\rightarrow{\mathcal {S}}^{n}$ in (6.2) and $n-s=i$ and $n-t=j$, we obtain the following result: If $K,L\in{\mathcal {S}}^{n}$, $0\leq p< n-1$ and $i\leq n-1\leq j\leq n$, then

6.5 $$\begin{aligned} \biggl(\frac{\widetilde{W}_{i}(\mathbf{I}_{p}(K\widehat {+}_{p}L))}{\widetilde{W}_{j}(\mathbf{I}_{p}(K\widehat{+}_{p}L))} \biggr)^{1/(j-i)} \leq \biggl(\frac{\widetilde{W}_{i}({\bf I}_{p}K)}{\widetilde{W}_{j}(\mathbf{I}_{p}K)} \biggr) ^{1/(j-i)}+ \biggl(\frac{\widetilde{W}_{i}({\bf I}_{p}L)}{\widetilde{W}_{j}({\bf I}_{p}L)} \biggr)^{1/(j-i)}, \end{aligned}$$

with equality if and only if $\mathbf{I}_{p}K$ and $\mathbf{I}_{p}L$ are dilates. Taking $j=n$ in (6.5) and noting that $\widetilde{W}_{n}(K)=\int_{S^{n-1}}\,dS(u)=n\omega_{n}$, (6.5) becomes the following inequality: If $K,L\in{\mathcal {S}}^{n}$, $0\leq p< n-1$ and $i\leq n-1$, then

$$\widetilde{W}_{i}\bigl(\mathbf{I}_{p}(K \widehat{+}_{p}L)\bigr)^{1/(n-i)} \leq\widetilde{W}_{i}({ \bf I}_{p}K)^{1/(n-i)}+\widetilde{W}_{i}( \mathbf{I}_{p}L)^{1/(n-i)}, $$

with equality if and only if $\mathbf{I}_{p}K$ and $\mathbf{I}_{p}L$ are dilates. □

We are now in a position to prove Theorem 5.3. The following statement is just a slight reformulation of it.

### Theorem 6.3

*Let* $K,L\in{\mathcal {S}}^{n}$. *If* $0\leq i< n $, $p< i-1$ *and* $s,t\in{\Bbb {R}}$ *satisfy* $s\geq1\geq t\geq0$, *then*

6.6 $$\begin{aligned} &\frac{1}{\widetilde{W}_{i}(K\breve{+}_{p} L)} \biggl(\frac{\widetilde{W}_{n-s}(\Psi_{p}(K\breve {+}_{p}L))}{\widetilde{W}_{n-t}(\Psi_{p}(K\breve{+}_{p}L))} \biggr)^{1/(s-t)} \\ &\quad\leq\frac{1}{\widetilde{W}_{i}(K)} \biggl(\frac {\widetilde{W}_{n-s}(\Psi_{p}K)}{\widetilde{W}_{n-t}(\Psi _{p}K)} \biggr) ^{1/(s-t)}+ \frac{1}{\widetilde{W}_{i}(L)} \biggl(\frac{\widetilde {W}_{n-s}(\Psi_{p}L)}{\widetilde{W}_{n-t}(\Psi_{p}L)} \biggr)^{1/(s-t)}, \end{aligned}$$

*with equality if and only if* $\Psi_{p}K$ *and* $\Psi_{p}L$ *are dilates*.

### Proof

From (3.3), we obtain

$$\frac{\rho(K\breve{+}_{p} L,\cdot)^{n-p-1}\ast\mu}{\widetilde{W}_{i}(K\breve{+}_{p} L)}=\frac{\rho(K,\cdot)^{n-p-1}\ast\mu}{\widetilde {W}_{i}(K)}+\frac{\rho(L,\cdot)^{n-p-1}\ast\mu}{\widetilde{W}_{i}(L)}. $$

Hence, from (4.6), we obtain

$$\frac{\rho(\Psi_{p}(K\breve{+}_{p}L),\cdot)}{\widetilde {W}_{i}(K\breve{+}_{p} L)}=\frac{\rho(\Psi_{p}K,\cdot)}{\widetilde{W}_{i}(K)}+\frac{\rho (\Psi_{p}L,\cdot)}{\widetilde{W}_{i}(L)}. $$

By (1.6), we have

6.7 $$\begin{aligned} \frac{\widetilde{W}_{n-s}(\Psi_{p}(K\breve{+}_{p}L))}{\widetilde {W}_{i}(K\breve{+}_{p} L)^{s}}=\frac{1}{n} \int_{S^{n-1}} \biggl(\frac{\rho(\Psi _{p}K,u)}{\widetilde{W}_{i}(K)}+\frac{\rho(\Psi_{p}L,u)}{\widetilde {W}_{i}(L)} \biggr)^{s}\,dS(u) \end{aligned}$$

and

6.8 $$\begin{aligned} \frac{\widetilde{W}_{n-t}(\Psi_{p}(K\breve{+}_{p}L))}{\widetilde {W}_{i}(K\breve{+}_{p} L)^{t}}=\frac{1}{n} \int_{S^{n-1}} \biggl(\frac{\rho(\Psi _{p}K,u)}{\widetilde{W}_{i}(K)}+\frac{\rho(\Psi_{p}L,u)}{\widetilde {W}_{i}(L)} \biggr)^{t}\,dS(u). \end{aligned}$$

From (6.7), (6.8) and Lemma 6.1, we obtain

$$\begin{aligned} &\frac{1}{\widetilde{W}_{i}(K\breve{+}_{p} L)} \biggl(\frac{\widetilde{W}_{n-s}(\Psi_{p}(K\breve {+}_{p}L))}{\widetilde{W}_{n-t}(\Psi_{p}(K\breve{+}_{p}L))} \biggr)^{1/(s-t)} \\ &\quad= \biggl( \frac{\int_{S^{n-1}} (\frac{\rho(\Psi _{p}K,u)}{\widetilde{W}_{i}(K)}+\frac{\rho(\Psi_{p}L,u)}{\widetilde {W}_{i}(L)} )^{s}\,dS(u)}{ \int_{S^{n-1}} (\frac{\rho(\Psi_{p}K,u)}{\widetilde {W}_{i}(K)}+\frac{\rho(\Psi_{p}L,u)}{\widetilde{W}_{i}(L)} )^{t}\,dS(u)} \biggr)^{1/(s-t)} \\ &\quad\leq \biggl(\frac{\int_{S^{n-1}} (\frac{\rho(\Psi_{p}K,u)}{\widetilde {W}_{i}(K)} )^{s}\,dS(u)}{ \int_{S^{n-1}} (\frac{\rho(\Psi_{p}K,u)}{\widetilde {W}_{i}(K)} )^{t}\,dS(u)} \biggr)^{1/(s-t)}+ \biggl(\frac{\int _{S^{n-1}} (\frac{\rho(\Psi_{p}L,u)}{\widetilde {W}_{i}(L)} )^{s}\,dS(u)}{ \int_{S^{n-1}} (\frac{\rho(\Psi_{p}L,u)}{\widetilde {W}_{i}(L)} )^{t}\,dS(u)} \biggr)^{1/(s-t)} \\ &\quad=\frac{1}{\widetilde{W}_{i}(K)} \biggl(\frac {\widetilde{W}_{n-s}(\Psi_{p}K)}{\widetilde{W}_{n-t}(\Psi _{p}K)} \biggr) ^{1/(s-t)}+ \frac{1}{\widetilde{W}_{i}(L)} \biggl(\frac{\widetilde {W}_{n-s}(\Psi_{p}L)}{\widetilde{W}_{n-t}(\Psi_{p}L)} \biggr)^{1/(s-t)}, \end{aligned}$$

with equality if and only if $\Psi_{p}K$ and $\Psi_{p}L$ are dilates.

Taking $s=n-j$ and $t=n-k$ in Theorem 6.3, Theorem 6.3 becomes Theorem 5.3 stated in Section 5. If $\Psi:\underbrace{{\mathcal {S}}^{n}\times\cdots\times{\mathcal {S}}^{n}}_{n-1}\rightarrow {\mathcal {S}}^{n}$ is the mixed intersection operator ${\bf I}:\underbrace{{\mathcal {S}}^{n}\times\cdots\times{\mathcal {S}}^{n}}_{n-1}\rightarrow{\mathcal {S}}^{n}$ in (6.6) and $j=n-s$ and $k=n-t$, we obtain the following result: If $K,L\in{\mathcal {S}}^{n}$, $0\leq i< n$, $p< i-1$ and $j\leq n-1\leq k\leq n$, then

6.9 $$\begin{aligned} &\frac{1}{\widetilde{W}_{i}(K\breve{+}_{p} L)} \biggl(\frac{\widetilde{W}_{j}({\bf I}_{p}(K\breve{+}_{p}L))}{\widetilde{W}_{k}({\bf I}_{p}(K\breve{+}_{p}L))} \biggr)^{1/(k-j)} \\ &\quad \leq \frac{1}{\widetilde{W}_{i}(K)} \biggl(\frac{\widetilde {W}_{j}({\bf I}_{p}K)}{\widetilde{W}_{k}(\mathbf{I}_{p}K)} \biggr) ^{1/(k-j)}+ \frac{1}{\widetilde{W}_{i}(L)} \biggl(\frac{\widetilde {W}_{j}({\bf I}_{p}L)}{\widetilde{W}_{k}({\bf I}_{p}L)} \biggr)^{1/(k-j)}, \end{aligned}$$

with equality if and only if $\mathbf{I}_{p}K$ and $\mathbf{I}_{p}L$ are dilates. Taking $k=n$ in (6.9) and noting that $\widetilde{W}_{n}(K)=\int_{S^{n-1}}\,dS(u)=n\omega_{n}$, (6.9) becomes the following inequality: If $K,L\in{\mathcal {S}}^{n}$, $0\leq i< n$, $p< i-1$ and $j\leq n-1$, then

6.10 $$\begin{aligned} \frac{\widetilde{W}_{j}({\bf I}_{p}(K\breve{+}_{p}L))^{1/(n-j)}}{\widetilde{W}_{i}(K\breve{+}_{p} L)}\leq\frac{\widetilde{W}_{j}({\bf I}_{p}K)^{1/(n-j)}}{\widetilde{W}_{i}(K)}+\frac{\widetilde {W}_{j}({\bf I}_{p}L)^{1/(n-j)}}{\widetilde{W}_{i}(L)}, \end{aligned}$$

with equality if and only if $\mathbf{I}_{p}K$ and $\mathbf{I}_{p}L$ are dilates. □

## Conclusions

In the present study, we first revised and improved the concepts of radial Blaschke addition and harmonic Blaschke addition in an $L_{p}$ space. Following this, we established Dresher’s inequalities (Brunn-Minkowski type) for the radial Blaschke-Minkowski homomorphisms with respect to the *p*-radial addition and the *p*-harmonic Blaschke addition.
